# Detection of Metastatic Tumor Cells in the Bone Marrow Aspirate Smears by Artificial Intelligence (AI)-Based *Morphogo* System

**DOI:** 10.3389/fonc.2021.742395

**Published:** 2021-09-27

**Authors:** Pu Chen, Run Chen Xu, Nan Chen, Lan Zhang, Li Zhang, Jianfeng Zhu, Baishen Pan, Beili Wang, Wei Guo

**Affiliations:** ^1^ Department of Laboratory Medicine, Zhongshan Hospital, Fudan University, Shanghai, China; ^2^ Department of Medical Development, Hangzhou ZhiWei Information Technology Co. Ltd., Hangzhou, China; ^3^ Department of Laboratory Medicine, Xiamen Branch, Zhongshan Hospital, Fudan University, Xiamen, China; ^4^ Department of Laboratory Medicine, Wusong Branch, Zhongshan Hospital, Fudan University, Shanghai, China

**Keywords:** bone marrow, metastatic cancer, artificial intelligence, *morphogo*, convolutional neural network

## Abstract

**Introduction:**

Metastatic carcinomas of bone marrow (MCBM) are characterized as tumors of non-hematopoietic origin spreading to the bone marrow through blood or lymphatic circulation. The diagnosis is critical for tumor staging, treatment selection and prognostic risk stratification. However, the identification of metastatic carcinoma cells on bone marrow aspiration smears is technically challenging by conventional microscopic screening.

**Objective:**

The aim of this study is to develop an automatic recognition system using deep learning algorithms applied to bone marrow cells image analysis. The system takes advantage of an artificial intelligence (AI)-based method in recognizing metastatic atypical cancer clusters and promoting rapid diagnosis.

**Methods:**

We retrospectively reviewed metastatic non-hematopoietic malignancies in bone marrow aspirate smears collected from 60 cases of patients admitted to Zhongshan Hospital. High resolution digital bone marrow aspirate smear images were generated and automatically analyzed by *Morphogo* AI based system. *Morphogo* system was trained and validated using 20748 cell cluster images from randomly selected 50 MCBM patients. 5469 pre-classified cell cluster images from the remaining 10 MCBM patients were used to test the recognition performance between Morphogo and experienced pathologists.

**Results:**

*Morphogo* exhibited a sensitivity of 56.6%, a specificity of 91.3%, and an accuracy of 82.2% in the recognition of metastatic cancer cells. *Morphogo*’s classification result was in general agreement with the conventional standard in the diagnosis of metastatic cancer clusters, with a Kappa value of 0.513. The test results between *Morphogo* and pathologists H1, H2 and H3 agreement demonstrated a reliability coefficient of 0.827. The area under the curve (AUC) for *Morphogo* to diagnose the cancer cell clusters was 0.865.

**Conclusion:**

In patients with clinical history of cancer, the *Morphogo* system was validated as a useful screening tool in the identification of metastatic cancer cells in the bone marrow aspirate smears. It has potential clinical application in the diagnostic assessment of metastatic cancers for staging and in screening MCBM during morphology examination when the symptoms of the primary site are indolent.

## Introduction

Metastatic carcinomas of bone marrow (MCBM) are defined as non-hematopoietic malignancies that metastasize to bone marrow. The incidence, clinical presentation and laboratory findings of metastatic carcinoma in the bone marrow are quite variable. The underlying process of metastasis is complex and contribute to significant clinical outcomes of cancer related death. The five key steps of metastasis include cascades of invasion, intravasation, circulation, extravasation, and colonization. Tumor cells may express some adhesion molecules that promote the transmigration to the marrow space and link them to the marrow stroma with subsequent engraftment (1, 2). In advanced stages, many of the commonly occurring solid tumors exhibit a high incidence of bone marrow involvement. These tumors include breast, prostate, lung and gastrointestinal tract cancers in adults and neuroblastoma in children. However, due to occulting and atypical clinical manifestations, bone marrow metastases can be easily missed or misdiagnosed, leading to higher mortality rates (3, 4). Bone marrow metastasis is initially presented before the primary tumor site in many advanced cancer patients (5). Therefore, early bone marrow metastasis identification is important for early cancer diagnosis and treatment.

Classification and differential counting of bone marrow cells are the fundamental steps of diagnostic hematology. The infiltration of cancer cells can cause bone marrow structure destruction and hematopoietic dysfunction, resulting in alterations in blood and bone marrow images (6, 7). In many circumstances, the identification of abnormal cells in bone marrow smears is the primary critical finding for the final diagnosis (8–10). Currently, methods for automatic differential counting of peripheral blood are readily available commercially. However, morphological assessment and differential counting of bone marrow smears are still performed manually. This procedure is tedious, time-consuming and laden with high inter-operator variation (11). It is even more challenging to identify occult metastatic cancer cells manually because they are not distributed evenly across the smear (12, 13). Most of them are located at the edges of slides beyond for recognition, leading to misdiagnosis in clinical practice (14). Furthermore, the distinction of poorly differentiated carcinomas from hematopoietic tumors, particularly acute leukemia or large cells lymphoma, is very difficult.

In recent years, deep neural networks have been proven useful in many medical image recognition tasks, such as diagnosis of diabetic retinopathy, and detection of cancer metastasis in lymph nodes (15). However, to our knowledge, there is no reliable method to identify metastatic carcinoma cells based on complete differential counting of entire bone marrow smears using a deep neural network. In this study, we present the results of using a deep convolutional neural network for automatic identification of bone marrow metastatic carcinoma cells.

Because of the lack of experienced pathologists, especially in rural hospitals, metastatic carcinoma cells can easily be missed. The objective of this study is to develop an algorithm to automatically screening whole bone marrow smears for metastatic carcinoma cells. To do so, we have created a well-annotated metastatic carcinoma cell clusters image dataset for deep neural network training in recognition of metastatic malignancy. Compared with other technologies, such as bone marrow biopsy, our approach is faster, labor-saving, and cost effective. The initial results are promising, and with the ongoing expansion of datasets and further training, our system would be more precise and clinically useful.

## Materials and Methods

### Patients

This study included all 60 patients admitted to Zhongshan Hospital, Fudan University with solid tumors and diagnosed with bone marrow metastasis between 1st March 2007 and 23rd October 2020. Inclusion criteria: Patients were diagnosed with bone marrow metastatic cancer if they met the diagnostic criteria mentioned on the expert consensus statement on clinical diagnosis and treatment of malignant tumor bone metastases. Bone marrow biopsy and immunohistochemistry (IHC) analysis were performed to confirm diagnosis. Exclusion criteria: Histopathology confirmed leukemia, lymphoma, myeloma and other hematopoietic malignancies; early tumor; and incomplete clinical and pathological data.

### Preparation of Bone Marrow Smear

Bone marrow biopsy needle (B65-01) was used for one-step sampling after local anaesthesia of the patient’s posterior superior iliac spine. 0.2 mL of bone marrow solution was extracted, and a bone marrow smear with uniform thickness was made immediately. After natural drying, Wright-Giemsa staining was performed for routine examination.

### Hardware

To obtain high-resolution images of bone marrow aspirate smears, a new automatic smear scanning device named *Morphogo* was developed. *Morphogo*’s hardware consisted of the preview cam, QR code printer, scanner and computer. The preview box is used to select the scanning area. The smears were loaded into the scanner in a tray that can hold up to 27 slides. A microscopy unit is installed in the scanner with a 40 × objective (Plan N 40 ×/0.65 FN22, resolution 0.42 μm, Olympus, Japan) and a 100 × objective (Plan N 100 ×/1.25 FN22, resolution 0.22 μm, Olympus, Japan), an oil-dropping unit, a light source unit and a camera with 4000 × 3000 pixels (E3ISPM12000KPA with 12MP 1/1.7”(7.40 × 5.55) SONY Exmor CMOC Sensor (ToupCam, China).

### Software

The software of *Morphogo* consists of three types of clients: the acquisition terminal, the review terminal and the consultation terminal. The acquisition terminal was based on a 27-layered convolutional neural network. After the scanner captured many small high-resolution images of the bone marrow smear, the acquisition terminal montaged them to create a full image of the bone marrow aspirate smear, localized the potential cancel cell clusters, extracted the key features and classified each cell cluster as carcinoma/non-carcinoma. The review terminal was used by the pathologists H1, H2 and H3 to re-label these pre-classified cell clusters.

### CNN Architecture and Training Details

The convolutional neural network of *Morphogo’s* acquisition terminal contains convolution layers, pooling layers, and fully connected layers. The convolution filter is used to modify or enhance the cell cluster image by emphasizing or removing certain features in image processing (16), including blurring, sharpening, embossing, edge detection, *etc*. The pooling layer reduces the dimensions of the data by combining outputs of a cluster of neurons at one layer into a single neuron in the next layer. It serves to reduce the spatial size of features, amount of computation in the network and number of parameters, and also controls overfitting and improves adversarial robustness (17). The fully connected layers were reformatted after several convolution and pooling layers. They will take high-level features generated by convolution or pooling layers and use them to classify the cell cluster images. The CNN used in this research contains 27 layers and is trained by a database of well-labelled metastatic carcinoma cells.

Pre-screening is first conducted by *Morphogo* in the saturation channel to extract areas with high saturation and aggregation characteristics as suspected cancer cell clusters. Only high saturation aggregations that have a surface area bigger than 60000 pixels and occupies at least 30% of the surface of the minimum bonding rectangle are selected as potential cancel cell clusters that need to be classified. Some single white blood cells or red blood cells clumps and staining artifacts as result of the preparative procedure are screened out and removed in this process.

**Figure 1:**
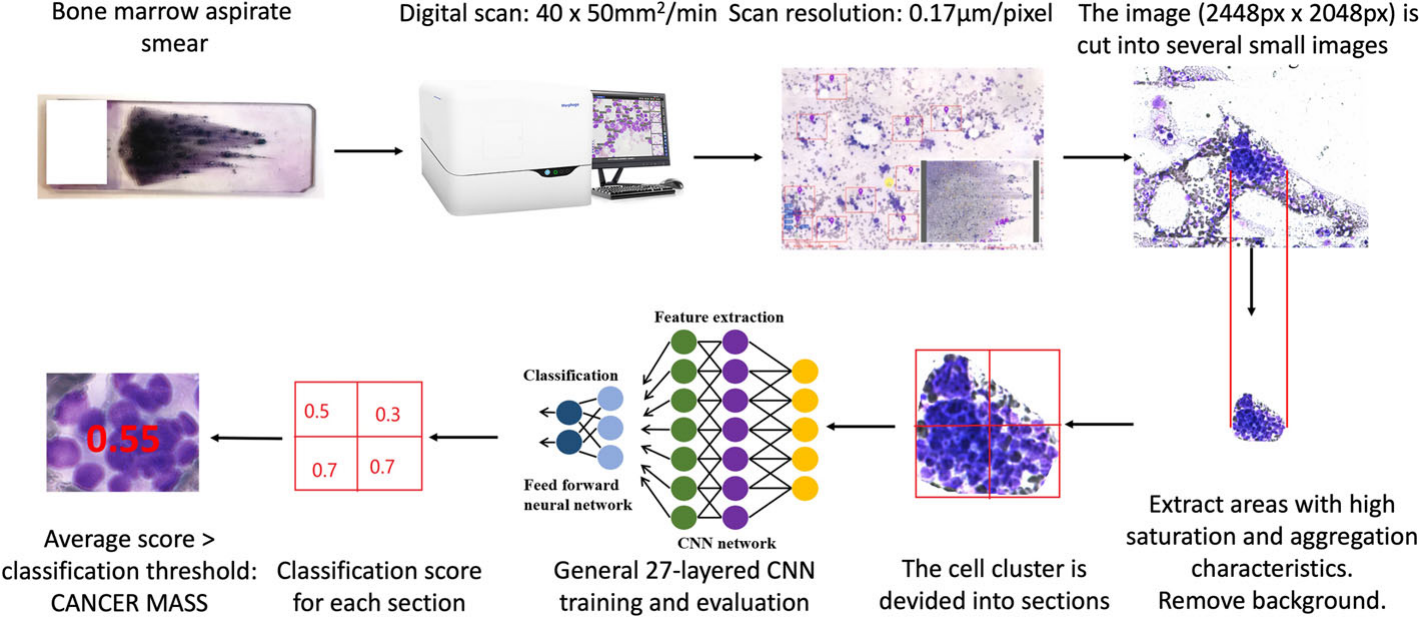
The workflow of identification metastatic atypical cancer cell clusters by *Morphogo* in bone marrow smears.

The workflow by which the *Morphogo* system identifies atypical cancer clusters is illustrated in **Figure 1**. Metastatic cancer clusters usually exhibited cohesive 3-dimensional architecture of aggregated large atypical cells with multinucleation, high nucleus/cytoplasmic ratio, hyperchromatic nuclei, irregular nuclei, variable nucleoli, atypical mitosis, polymorphisms and sometimes vacuolated cytoplasm. During the training of the deep Convolutional Neural Network (CNN), the cell cluster is divided into several small sections of fixed size. Each section’s probability of being a cancer cell cluster is calculated in terms of scores. If the average score of all sections is greater than the classification threshold, it is considered cancer mass. The CNN algorithms are trained and fine-tuned for accuracy, and the final *Morphogo* software is used to classify the cell clusters at a high velocity.

### Training and Validation Cohort

20748 cell cluster images were collected from 50 MCBM patients and randomly assigned to the training and validation sets according to a ratio of 0.8:0.2. The classification of each cell was annotated once by an experienced pathologist. The training set consists of 16598 cell cluster images and was used to train *Morphogo’s* model for instance segmentation task. During the model training, we used group normalization and stochastic gradient descent optimizer. Random noise, Gaussian blur, rotation, contrast and color shift were also used as means for data augmentation. The remaining 4150 cell cluster images were used as the validation set to evaluate the performance of *Morphogo* during training with different hyperparameter values. The validation set was also used to detect overfitting during the training stages. The training of *Morphogo* model was run on a server equipped with Intel Core i9 10,900X, 16G × 4 ADATA DDR4, NVIDIA GeForce RTX 2080 Ti cards, and CUDA Version 10.2. After repeated iterative training, an optimal algorithm for cell classification was obtained and internally verified.

### Test Cohort

The test set is used to evaluate the final carcinoma cell cluster recognition performance of *Morphogo* compared to experienced pathologists. It consists of 5,469 cell cluster images collected from 10 MCBM patients. The clusters in the training set, validation set, and test set are all different. The aim is to provide an unbiased evaluation of final model performance as co-occurrence might falsely improve the apparent prediction accuracy of the classification model. The cell classification was annotated by three experienced pathologists. To avoid the potential discrepancy, only over 2/3 consensus was regarded as a valid diagnosis. About 47% of the positive cases were confirmed by a full consensus and 90% of the negative cases achieved full consensus.

This final diagnosis was correlated with clinical and radiological findings and served as the standard for evaluation.

### Statistical Analysis

After the neural network model was fully trained, the ability of the model to classify and detect metastatic carcinoma cells was evaluated using the test set in terms of sensitivity, specificity, accuracy, positive predictive value and negative predictive value at different classification thresholds. Kappa and ICC for consistency evaluation of measurement methods were performed by SPSS v22.0 (IBM SPSS Statistics, IBM, Chicago, USA). The interpretation of K statistic is as follows: K is less than 0, inconsistent; K = 0-0.20, slightly consistent; K = 0.21-0.40, fair consistent; K = 0.41-0.60, moderately consistent; K = 0.61-0.80, substantially consistent; K = 0.81-1.0, almost consistent. We estimated 95% confidence intervals (CI) for Kappa statistics. The ROC curve was plotted by MedCalc 19.7.2 and analyzed by Z test. The difference between the two groups was analyzed by student’s t test, *P* ≤ 0.05 was considered statistically significant.

## Results

### Comparison of the Diagnostic Performance of *Morphogo* and Pathologists

We chose two critical classification thresholds. One threshold is 0.426 for high specificity classification and the other one is 0.046 for high sensitivity classification. The sensitivity, specificity, accuracy of *Morphogo* were compared to those of the three pathologists at different thresholds. *Morphogo* had a sensitivity of 56.6% when the threshold is set to 0.426, which was lower than the pathologists (78.3%, 83.5% and 84.9%). As displayed in **Table 1**, the specificity of *Morphogo* was 91.3%, not significantly different from the pathologists (97.5%, 97.2% and 94.9%). The positive predictive value and negative predictive value of *Morphogo* were 69.5% and 85.7%, respectively. The classification accuracy of *Morphogo* was 82.2%, which was similar to the pathologists (92.5%, 93.6% and 92.2%). When the classification threshold is adjusted to 0.046, the sensitivity of Morphogo increased considerably to 89.4%, whereas the specificity and the accuracy decreased to 65.5% and 70.1%, respectively.

**Table 1:** The sensitivity, specificity, positive predictive value, negative predictive value of *Morphogo* and pathologist H1, H2 and H3.

Pathologist						
	Sample size	Sensitivity	Specificity	PPV	NPV	Accuracy
**H1**	5469	0.783	0.974	0.915	0.928	0.925
**H2**	5469	0.835	0.972	0.912	0.944	0.937
**H3**	5469	0.849	0.949	0.853	0.947	0.923
**Morphogo**						
**Classification threshold**	**Sample size**	**Sensitivity**	**Specificity**	**PPV**	**NPV**	**Accuracy**
**0.426**	5469	0.566	0.913	0.695	0.857	0.822
**0.045**	5469	0.894	0.655	0.476	0.946	0.717
**0.025**	1147 (cases with two consensus)	0.909	0.232	0.682	0.584	0.668
**0.102**	4295 (cases with three consensus)	0.910	0.778	0.429	0.979	0.798

PPV, Positive predictive value; NPV, Negative predictive value.

**Figure 2:**
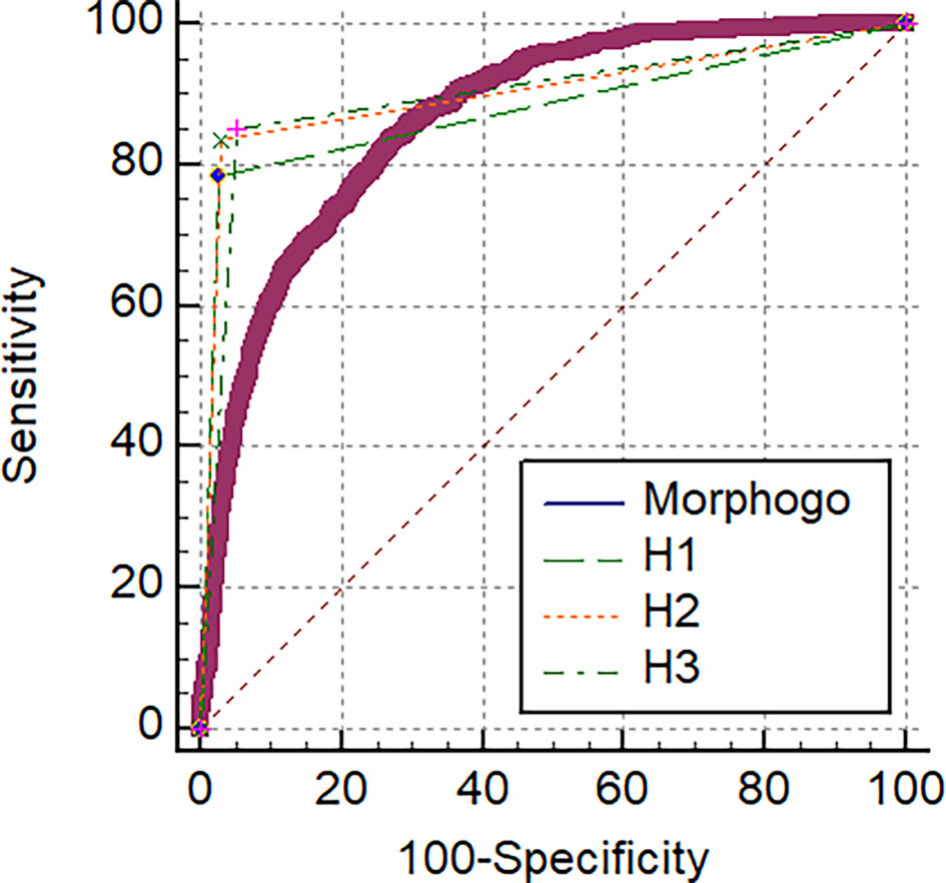
The correlation of ROC curve between *Morphogo* and pathologists.

As shown in **Figure 2** and **Table 2**, the area under the curve (AUC) for *Morphogo* to diagnose the cancer cell clusters was 0.865 (threshold = 0.426), which was lower than the AUC of pathologist H1, H2 and H3, and the difference was statistically significant (*Z* > 1.96, *P* < 0.05). The optimal diagnostic cut-off point of the *Morphogo* system was 0.485, and the Youden index was 0.808. This data indicates that *Morphogo* was lower in the recognition and diagnosis of cancer cluster cells than pathologists. As illustrated in **Table 3**, the time required for the *Morphogo* to identify and count metastatic cancer clusters of digital microscope photos is 8.86 ± 0.00 s/1000 images, which was significantly less than the pathologists. The above results suggest that the combined diagnosis of pathologists and *Morphogo* system may improve the detection rate of cancer cell clusters more efficiently and accurately, and greatly save the time of pathologists.

**Table 2:** Comparison of diagnostic values of *Morphogo* (classification threshold = 0.426) and pathologists H1, H2 and H3.

Method	AUC	SE	95%CI	Z value	*P* value
***Morphogo***	0.865	0.00526	0.855~0.874	/	/
**H1**	0.879	0.0056	0.870~0.888	2.107	0.0351
**H2**	0.904	0.00509	0.896~0.911	5.544	< 0.0001
**H3**	0.899	0.00506	0.891~0.907	5.055	< 0.0001

**Table 3:** The time (s/1000 images) required for *Morphogo* and pathologists to identify and count metastatic cancer clusters from digital microscope imagines on the marrow smears.

Method	Time (s/1000 images)	*P* value
***Morphogo***	8.86 ± 0.00	/
**pathologists**	5200 ± 458.26	0.0001

### Comparison of the Inter-Rater Agreement of *Morphogo* and Pathologists

*Morphogo* group was in general agreement with the gold standard in the diagnosis of metastatic cancer clusters, with a Kappa value of 0.513. Pathologist H1, H2, and H3 were in good agreement with the gold standard in the diagnosis of metastatic cancer clusters, with Kappa values of 0.796, 0.830 and 0.799, respectively. Moreover, *Morphogo* and Pathologists H1, H2, H3 had a reliability coefficient (ICC) of 0.827 ± 0.007 (*F* value = 5.791, *P* value = 0), indicating that they had a high degree of consistency in identifying cancer cell clusters **(** **Table 4** **).**

**Table 4:** Evaluation of the consistency between the pathologists/Morphogo (classification threshold = 0.426) and the gold standard in terms of Cohen kappa coefficient.

Method	Kappa	*P* value
***Morphogo***	0.513	0.000
**H 1**	0.796	0.000
**H2**	0.830	0.000
**H 3**	0.799	0.000

### Identification of Cancer Cell Morphology by *Morphogo*

**Figure 3:**
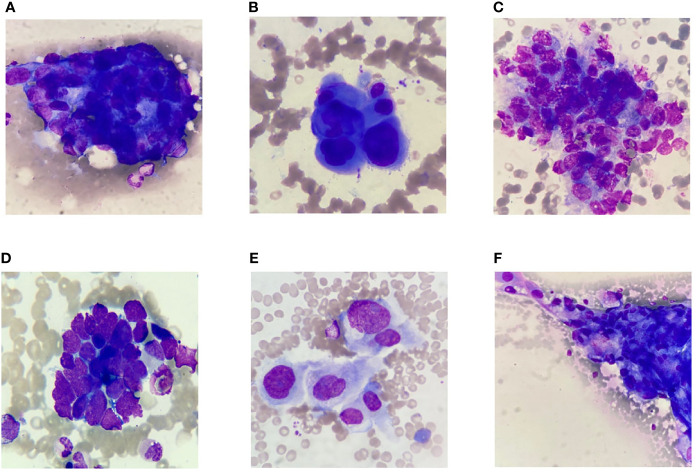
Selected representative images of bone marrow smears extracted from digital histopathological microscopic scans. **(A)** Gastric carcinoma. **(B)** Breast carcinoma. **(C)** Prostate carcinoma. **(D)** Lung small cell carcinoma. **(E)** Cholangiocarcinoma. **(F)** Lung adenocarcinoma.

Metastatic cancer cells were found in 60 patients with MCBM after biopsy. Clustered and/or scattered cancer cells were found in the feather edge tail of the bone marrow smears. Good quality slide/sample preparation plays a pivotal role in good detection and subsequent classification of marrow cells and metastatic cells using digital imaging. The abnormal morphological characteristics of cancer cells are exhibited as the following criteria: the cells sizes of cancer cells are essentially larger than that of blood cells; the nuclei are hyperchromatic by dark blue stained and convoluted; the nucleoli are prominent; the cytoplasm is rich with degenerative vacuoles, and apoptosis and atypical mitosis are easily appreciated. The cancer cells are usually distributed in cohesive clumps with 3D architecture and the cell boundaries between cells are not clear. The hematopoietic cells and reactive lymphocytes, plasma cells and macrophages are seen in the background. **(** **Figure 3** **).**

Adenocarcinomas contributed the majority (54/of 60 cases) of metastatic neoplastic lesions. Metastatic adenocarcinoma from the GI tract formed the majority of cases (23/60), followed by breast cancer (11/60), lung cancer (10/60), prostate carcinoma (7/60) and undifferentiated malignancy (4/60). Metastatic adenocarcinoma cells from gastrointestinal tumor had abundant cytoplasm and large vesicular nuclei in loose clusters and groups. Metachromatic cytoplasmic granules were present in some cases **(** **Figure 3A** **).** Metastasis from a case of ductal carcinoma breast showed cohesive clusters of cells with moderately pleomorphic overlapping nuclei **(** **Figure 3B** **).** Metastatic germ cell tumor cells from a case of testicular mass had clusters of moderately pleomorphic cells with discernible cytoplasm. Lymphocytes were also present **(** **Figure 3C** **).** Tight clusters of hyperchromatic cells with scanty cytoplasm, nuclear molding with “salt and pepper” like chromatin were seen in small cell carcinoma from the lung (**Figure 3D**). The abundant eosinophilic cytoplasm, polygonal shape and large vesicular nuclei with prominent central nucleoli are characterized as hepatocellular carcinoma. Some of the tumor cells were arranged in sheets and clusters with acinar pattern or in trabecular pattern. Eosinophilic intranuclear inclusions were also noted **(** **Figure 3E** **).** Adenosquamous carcinoma had sheets of adenocarcinoma cells characterized by mildly pleomorphic cells with a moderate amount of cytoplasm and discretely present malignant squamous cells with hyperchromatic nuclei and abundant glassy to blue cytoplasm. Some of the adenocarcinomatous cells were tightly pressed against each other with vacuolated cytoplasm and complex nuclear features. Nucleoli were prominent in high-grade tumors **(** **Figure 3F** **).**

## Discussion

This AI-aided classification model might be applied in different clinical situations by adjusting the classification threshold. A high threshold indicates high specificity, which might be used for the diagnosis of patients with low possibility to develop metastatic carcinomas and very likely to be true negative (*i.e*. >90%). When the threshold is reduced, the sensitivity is increased considerably. This type of model could be used in patients with high possibility to have metastatic carcinomas to avoid misdiagnosis. In this case, *Morphogo* has a higher possibility to find metastatic cell clusters by screening the whole smear, even at the far end of the slide, an area that is easily ignored in clinic practice, especially in rural hospitals where experienced pathologists are badly demanded. In fact, **Table 1** shows that the model with high sensitivity (threshold = 0.0459) exhibits a sensitivity of 89.4%, higher than pathologist H1 (78.3%) and H2 (83.5%), with acceptable specificity and comparable NPV. Although the accuracy of the model still needs to be improved to meet the clinical practice requirements, interestingly, when we performed further data analysis using better qualified testing data, the performance of *Morphogo* improved. Out of all the 5469 samples in test set, only 47% of the positive cases were confirmed by a full consensus, which indicates that the classification of carcinoma cell clusters is highly subjective. We chose 4295 cases that were confirmed by a full consensus to test the performance of *Morphogo*. We found that the performance of *Morphogo* was significantly improved (threshold = 0.101532263157895, sensitivity =91.0%, specificity = 77.8%). This suggests that, if better qualified training dataset is added to train the algorithm, *Morphogo* has great potential to improve its accuracy.

Bone marrow metastasis has a profound impact on the prognosis and treatment of advanced stages of cancer patients (18). The incidence of bone marrow metastasis varies depending on the type of tumor and the length of the disease, and are more common in patients with prostate, breast, lung, or gastric cancer and neuroblastoma (19–21). Radiological examinations using computed tomography and positron emission tomography (PET-CT) or magnetic resonance imaging (MRI) are the most commonly used non-invasive methods for diagnosing BM metastases in cancer; however, bone metastases from malignancy may still be missed (22, 23). More recently, high sensitivity disease detecting methods including tumor cell isolation and cell free tumor DNA detection by next generation sequencing (NGS) test have increased the incidence of minimal morphologically occult peripheral blood or bone marrow detected of metastatic neoplasms. Mapping the metastatic cancer cascade onto ctDNA using genetic and epigenetic clonal tracking is a powerful tool to practice precision medicine and target therapy.

Evaluation of bone marrow metastasis is important for the initial staging of cancer and ongoing monitoring residual disease after treatment and predicting relapse. However, previous methods may not be a complete substitute for microscopic examination of bone marrow, especially in cases of suspected bone marrow metastases from malignancies of an unknown primary site. When the primary tumor site is unknown, the detection and identification of malignant cells in bone marrow may help clarify the primary source and prevent additional unnecessary diagnostic procedures (24).

In patients with clinical suspicion of advanced cancer, the bone marrow samples are great resources to rule out metastasis. Usually, several bone marrow smears, as well as core biopsy, are collected for analysis. Morphological evaluation of bone marrow aspiration and biopsy is an important procedure to diagnose metastatic cancer in patients with evidence of bone marrow invasion (3). Bone marrow aspirate smears are readily applied to evaluate MCBM. However, a throughout microscopic screening is still limited by the technical challenges. The main steps of the pathological analysis of bone marrow smears are as follows: observe the cell structure under the microscope at low magnification, assess the cell morphology at high magnification, and finally establish a diagnosis or make therapeutic decisions. To make a definitive diagnosis of bone marrow metastases, it is necessary to find the bone marrow metastatic tumor cells and then figure out the tumor origin based on selected panel of immunohistochemistry on marrow core or tissue biopsy.

Furthermore, the relation between bone niche and immune system in physiological and pathological activities is also an important mechanism for metastatic carcinoma. To our knowledge, bone marrow smear cytomorphology technology has not been used for osteoimmunology research. Skeletal metastasis and further intramedullary metastasis are sometimes the only early metastatic lesions of some solid tumor such as prostate cancer and breast cancer. In particular, metastasis in the bone marrow niche is considered to be an important transfer station for further distant metastasis. The microenvironment of the bone marrow niche decides when these cancer cells might furtherly metastasize to other organs. As Antonio et al. mentioned, a bidirectional process between cancer cells and bone niche could explain a possible both locoregional and systemic cancer control with the immune system serve a bridge between them (25). To test this hypothesis, bone marrow from model animals could be collected at different stages of carcinoma development until metastasis happens. Changes in the nucleated cells in bone marrow should be tested by both molecular and morphological technologies. The correlation of the change of RANK/RANKL signaling pathway and metastatic carcinoma from solid cancers, such as breast cancer, could be studied by examining patient samples with both molecular and morphological technologies.

Depending on the degree of complexity of each case, this analysis process is variable taking from hours to even days. Training an excellent pathologist requires the accumulation of over one hundred thousand photograph examination experiences. Currently, the shortage of experienced cytopathologists and the overloaded work is far from meeting patients’ diagnosis needs (11). Therefore, it is crucial to develop a more reliable objective and automated analysis system.

In recent years, deep learning methods have driven great success in the computer vision field. With the development of photomicrography and whole section scanning technology (26, 27), pathological slide sections can be saved as digital images, allowing the application of computer vision techniques in the field of pathology. Some histopathological auxiliary diagnosis methods based on deep learning have emerged. These methods can assist pathologists in disease diagnosis, solve the time-consuming problems and hidden dangers of omissions in clinical examinations. Furthermore, it improves the efficiency and quality of pathological diagnosis, and alleviate the plight of insufficient resources of some procedures, such as chest X-ray criteria (23), diagnosis of cancer with lymph node metastasis (24), and fundus photography (26), *etc*.

Commercial computer-aided diagnosis systems can be used for clinical identification of peripheral blood samples (27). However, there are still many challenges in the automatic identification and detection of cancer cell clusters in bone marrow smear. Bone marrow smears have more significantly diversified higher cell density than peripheral blood smear, more cohesive aggregation of samples, and focal plane fluctuation of multiple layers of cells. *Morphogo* system was well designed to successfully overcome these technical difficulties and can automatically identify bone marrow smears (28). By identifying and accumulating observed information from smears, this artificial intelligent-based model will improve the efficiency in recognition and count different cell types in bone marrow smears.

Our previous study has demonstrated the successful classification of bone marrow cells in the spectrum of lineage and maturation. The final performance of cell classification contained 230 bone marrow smears and 65986 nucleated cells (28). The current study is an expanded-out system in the recognition of non-hematopoietic cells.

Bone marrow metastasis of cancer cells may also be mimicking high-grade hematologic malignancy. Identifying the primary site of origin for metastases is dependent on the morphologic findings, immunohistochemical profile, and most importantly, clinical history. In clinical practice, metastasis solid tumor cells are occasionally found in bone marrow smear during morphology examination because of hematological manifestations such as anemia, especially when the symptoms of the primary site are indolent. Under this circumstance, the carcinoma cell clusters in bone marrow are the only first clinic evidence for metastatic carcinoma of bone marrow (MCBM). Afterwards, subsequent technologies such as computer-tomography (CT) scans, serum assessment, and biopsy *etc*. can be implemented to help find the original site (29).

Usually, epithelial tumors (carcinomas) tend to form groups of cohesive cells with a desmoplastic stromal reaction that are typically easy to distinguish from normal hematopoietic cells. On rare occasions, myeloid neoplasia can exhibit some unusual histologic patterns with cellular features mimicking metastatic carcinoma, which may show clusters of infiltration of large and atypical immature cells with basophilic cytoplasm and cytoplasmic vacuoles (30). Diagnostic pitfalls of the present study may include sampling discrepancy due to marrow fibrosis, hemodilution, and/or repeated necrotic aspirations misinterpreted as cancer, *etc*. Thus, all the marrow smears must be interpreted in correlation with the relevant clinical radiological findings. In the challenging cases, an elective radiology guided bone marrow core biopsy of the lesion with selected immunophenotypical analysis is needed in order to render a precise diagnosis.

In the present study, the trend of consistency of *Morphogo* automatic recognition system exhibits a good agreement with those of pathologists. The approaching diagnostic performance of sensitivity, accuracy, PPV, NPV, and AUC based on *Morphogo* system are still suboptimal comparing with the consent of a group of experienced pathologists. As a screening tool, we expected *Morphogo* system will improve the workflow of bone marrow smears examination in the clinical practice of diagnostic metastatic cancers. It is a useful ancillary test, but not a final diagnosis. The final interpretations still require clinical radiological correlation and judgement by well-trained pathologists.

The limitation of this pilot study is based on a single institutional retrospective study with potential selection bias of the cases. Therefore, the clinical utility requires multicenter validation of the *Morphogo* system for further improvement of the algorithm, especially to distinguish the hematopoietic islands, megakaryocytes clumps, stromal tissue cells and dye residues artifact that are prone to miscalculated as cancer cell clusters. A large scale multicenter collaborative initiative to prospective screen of bone marrow specimen using *Morphogo* system is still going on and will be reported in the near future.

## Conclusion

In summary, we applied an automated system *Morphogo* to identify metastatic atypical cancer cell clusters in bone marrow smears. It has the potential to assist the clinical application in the diagnosis of metastatic malignancy.

## Data Availability Statement

The raw data supporting the conclusions of this article will be made available by the authors, without undue reservation.

## Ethics Statement

The use of human samples was approved by the Ethics Committee of Zhongshan Hospital, Fudan University. The patients provided their written informed consent to participate in this study.

## Author Contributions

PC was the first author, major contributor of the research, experimental design and bone marrow aspirate smear diagnosis. RC was the second author, helped in the MCBM detection algorithm development, data analysis and manuscript writing. NC, LaZ, LiZ, and JZ assisted in bone marrow aspirate smear sample collection and diagnosis. BP was the scientific advisor. BW is the minor corresponding author and WG is the major corresponding author. All authors contributed to the article and approved the submitted version.

## Funding

This work was supported by grants from the National Natural Science Foundation of China (81972000, 81772263, 81902139), the Constructing Project of Clinical Key Disciplines in Shanghai (shslczdzk03302), the Key Medical and Health Projects of Xiamen (YDZX20193502000002), and Shanghai Medical Key Specialty (ZK2019B28).

## Conflict of Interest

RC is employed by Hangzhou ZhiWei Information Technology Co. Ltd.

The remaining authors declare that the research was conducted in the absence of any commercial or financial relationships that could be construed as a potential conflict of interest.

## Publisher’s Note

All claims expressed in this article are solely those of the authors and do not necessarily represent those of their affiliated organizations, or those of the publisher, the editors and the reviewers. Any product that may be evaluated in this article, or claim that may be made by its manufacturer, is not guaranteed or endorsed by the publisher.
